# Efficacy of dipeptidyl peptidase-4 inhibitor linagliptin in patients with type 2 diabetes undergoing hemodialysis

**DOI:** 10.1186/s13098-015-0043-2

**Published:** 2015-05-17

**Authors:** Yuichi Terawaki, Takashi Nomiyama, Hiroyuki Takahashi, Yoko Tsutsumi, Kunitaka Murase, Ryoko Nagaishi, Makito Tanabe, Tadachika Kudo, Kunihisa Kobayashi, Tetsuhiko Yasuno, Hitoshi Nakashima, Toshihiko Yanase

**Affiliations:** Department of Endocrinology and Diabetes Mellitus, School of Medicine, Fukuoka University, 7-45-1 Nanakuma, Jonan-ku, Fukuoka, 814-0180 Japan; Department of Endocrinology and Diabetes Mellitus, Fukuoka University Chikushi Hospital, 1-1-1 Zokumyouin, Chikushino, Fukuoka 818-0067 Japan; Division of Nephrology and Rheumatology, Department of Internal Medicine, Fukuoka University, School of Medicine, 7-45-1 Nanakuma, Jonan-ku, Fukuoka, 814-0180 Japan

**Keywords:** Type 2 diabetes, Hemodialysis, DPP-4 inhibitor, Linagliptin, Oxidized low-density lipoprotein

## Abstract

**Background:**

Incretin therapy is feasible in patients with type 2 diabetes mellitus undergoing hemodialysis (HD). However, few studies have examined the safety and efficacy of this therapeutic approach in patients with diabetes and renal impairment. Here, we examined glycemic control and the anti-oxidative-stress effects of the dipeptidyl peptidase (DPP)-4 inhibitor linagliptin in patients with type 2 diabetes undergoing HD.

**Methods:**

Thirty-five patients with type 2 diabetes undergoing HD (including 13 insulin-treated patients) were switched from ongoing therapy to linagliptin (5 mg, once daily). Levels of fasting blood glucose, C-peptide immunoreactivity (CPR), glycated albumin, B-type natriuretic peptide, oxidized low-density lipoprotein (oxLDL), high-sensitivity C-reactive protein, 8-hydroxy-2′-deoxyguanosine (8OHdG), body mass index, blood pressure, and other biologic characteristics (liver function, renal function, lipid profile) were determined before and 3 months after linagliptin treatment. Patients were classified into insulin-treated and non-insulin groups.

**Results:**

With the exception of levels of total bilirubin, aspartate aminotransferase, and CPR, none of the patients exhibited changes in glucose metabolism after switching to linagliptin treatment. However, oxLDL levels were decreased significantly by linagliptin therapy in the non-insulin-treated group despite the absence of changes in glycemic control.

**Conclusion:**

Linagliptin can decrease serum levels of oxLDL in patients with type 2 diabetes undergoing HD independent of its glucose-lowering effect.

## Background

Diabetes mellitus is a multifactorial progressive disease accompanied by the development of systematic vascular complications, one of the most important of which is diabetic nephropathy. Diabetic nephropathy results in severe renal failure, which necessitates hemodialysis (HD). Indeed, diabetic nephropathy has been the leading cause of the requirement for HD in Japan since 1998. Controlling glucose levels in the blood of diabetic patients with severe renal failure is difficult because of: frequent hypoglycemia; restrictions in the use of anti-diabetic agents; the instability of glucose, insulin, and drug metabolites on the days when the patient does not receive HD.

Previously, we have reported the efficacy of incretin therapy using continuous monitoring of glucose in patients with type 2 diabetes undergoing HD [1]. However, one of the most important causes of death in patients undergoing HD is cardiovascular disease. Lowering the glomerular filtration rate in patients with chronic kidney disease increases oxidative stress [2]. This action increases the risk of cardiovascular disease, primarily through inactivation of nitric oxide and production of oxidized low-density lipoprotein (oxLDL) [3]. Thus, patients with type 2 diabetes undergoing HD must be administered anti-diabetic agents with associated anti-oxidative and vascular-protective effects.

Incretins such as glucagon-like peptide (GLP)-1 and glucose-dependent insulinotropic polypeptide have been reported not only to act on pancreatic β-cells to stimulate glucose-responsive insulin secretion, but also to provide tissue-protective effects beyond lowering of blood glucose [4]. The dipeptidyl peptidase (DPP)-4 inhibitor linagliptin has been approved for use in patients with declining renal function at the same dose as that administered to patients with normal renal filtration owing to its primarily hepatobiliary route of elimination from the body. Furthermore, the safety and efficacy of linagliptin in patients with diabetes and severe renal impairment have been reported [5]. Interestingly, a retrospective analysis revealed that linagliptin reduced cardiovascular events compared with other glucose-lowering agents [6]. Several studies have investigated the vascular-protective effects of linagliptin *via* incretin-dependent and incretin-independent mechanisms. These effects include anti-oxidative stress [7], inhibition of advanced glycation end-products (AGE) and their receptor (RAGE) axis [8], inhibition of vascular DPP-4 activity [9], and cardioprotection [10]. Furthermore, we have reported that linagliptin attenuates neointima formation after vascular injury through its anti-oxidative-stress effects [11]. Thus, these data suggest the anti-oxidative stress and vascular-protective effects of linagliptin.

However, there are no reports describing the anti-oxidative stress and vascular-protective effects of linagliptin in patients with type 2 diabetes undergoing HD. In the present study, we examined glycemic control and a marker of the anti-oxidative-stress effect of linagliptin in patients with type 2 diabetes undergoing HD.

## Methods

Thirty-five Japanese patients with type 2 diabetes undergoing HD (including 13 insulin-treated patients) aged 44–82 years were recruited to the study. Patients receiving anti-diabetic treatment were switched to linagliptin (5 mg, once daily) for 3 months. Some insulin-treated patients were hospitalized and switched to linagliptin, as reported previously [1]. Patients with a history of type 1 diabetes and diabetic ketoacidosis, impairment of intrinsic insulin secretion (fasting serum C-peptide immunoreactivity (CPR) < 2.0 ng/dL), severe cardiac disease (New York Heart Association grade ≥ III), or severe liver disease were excluded.

The following efficacy parameters were examined before and 3 months after treatment: fasting blood glucose (BG), CPR, glycated albumin (GA), B-type natriuretic peptide (BNP), oxLDL, high-sensitivity C-reactive protein (hsCRP), 8-hydroxy-2′-deoxyguanosine (8OHdG), body mass index, blood pressure, and other biological examinations (total protein, albumin, aspartate aminotransferase (AST), alanine aminotransferase (ALT), γ-glutamyl transpeptidase (γGTP), total bilirubin, alkaline phosphatase (ALP), lactate dehydrogenase (LDH), blood urea nitrogen (BUN), creatinine, total cholesterol, low-density lipoprotein-cholesterol (LDL-C), high-density lipoprotein-cholesterol (HDL-C), triglyceride). GA is a more reliable marker of glycemic control than glycated hemoglobin in patients with renal failure [12], so GA was measured as a marker of glycemic control in the present study. Blood samples were taken before the start and 3 months after linagliptin treatment when patients visited the clinic to receive HD. Serum levels of oxLDL (normal range for males < 45 years and females < 55 years: 46–82 U/L; normal range for males ≥ 45 years and females ≥ 55: 61–105 U/L) and 8OHdG were measured using an enzyme immunoassay at SRL Inc. (Tokyo, Japan). Other parameters were measured by staff in relevant departments within each hospital.

Baseline characteristics of the 35 patients are shown in Table 1. Mean duration of diabetes was 20.1 ± 1.8 years. Mean duration of HD was 3.5 ± 0.4 years. Of these 35 patients, seven patients had not received anti-diabetic drugs, eight patients received incretin therapy (four received DPP-4 inhibitors and four received liraglutide), eight patients received other anti-diabetic drugs, and 13 received insulin therapy (17.4 ± 2.3 U/day). None of the patients were treated with an anti-glutamic acid dehydrogenase antibody and none had a history of ketoacidosis. All patients were treated thrice weekly for 4–5 h with a bicarbonate dialysate containing 100 mg/dL of glucose. Eight patients were taking statins at baseline, and continued taking them during the study period. No patient received new anti-diabetic and anti-dyslipidemia agents during the study period.

**Table 1:** Patient characteristics at baseline

Parameters	Mean ± SE
Age (years)	65.4 ± 1.9
Male/female	15:20
Duration of diabetes (years)	20.1 ± 1.8
Duration of HD (years)	3.5 ± 0.4
Before treatment (n)	None: 7
	α-Glucosidase inhibitors: 5
	Sulfonylureas: 2
	Vildagliptin: 2
	Alogliptin: 2
	Liraglutide: 4
	Insulin: 13
	Statins: 9

HD, hemodialysis

All patients provided written informed consent to participate in this study. The protocol was approved by the Ethics Committees of Fukuoka University Hospital (Fukuoka, Japan). The study was carried out in accordance with the ethical principles of the Declaration of Helsinki (1964) amended in Edinburgh in 2000. The study protocol was registered as UMIN (ID: 000007716).

Paired *t-*tests were undertaken for statistical analyses as appropriate. *P <* 0.05 was considered significant. Results are the mean ± standard error of the mean.

## Results

No episodes of severe hyperglycemia, ketosis, severe nausea, or other adverse effects were observed in patients at any time during linagliptin treatment. Laboratory data before and after treatment in all patients are shown in Table 2. Serum levels of AST and total bilirubin were decreased significantly by linagliptin treatment, but these changes were within the normal range and not clinically significant. Serum levels of CPR were increased significantly by linagliptin treatment. In addition, there were no differences in levels of BG, GA, BNP, oxLDL, hsCRP, and 8OHdG, or lipid profiles, measured before and after treatment for all patients.

**Table 2:** Comparison of all patients before and after treatment

Parameter	Before	After
BMI (kg/m^2^) *n* = 30	23.0 ± 0.8	23.0 ± 0.8
Systolic blood pressure (mmHg) *n* = 31	150.0 ± 5.0	159.4 ± 4.2
Diastolic blood pressure (mmHg) *n* = 31	73.1 ± 3.0	76.3 ± 2.6
Total protein (g/dL)	6.5 ± 0.1	6.6 ± 0.1
Albumin (g/dL) *n* = 34	3.7 ± 0.1	3.8 ± 0.1
AST (U/L)	15.1 ± 0.8	13.4 ± 0.8^*^
ALT (U/L) *n* = 34	12.0 ± 1.0	10.1 ± 1.1
γGTP (U/L)	22.5 ± 2.5	23.4 ± 4.2
Total bilirubin (mg/dL) *n* = 29	0.35 ± 0.02	0.28 ± 0.02^*^
ALP (U/L) *n* = 34	257.4 ± 23.6	265.1 ± 22.0
LDH (U/L) *n* = 34	189.6 ± 5.9	183.3 ± 5.7
BUN (mg/dL)	54.7 ± 1.7	54.0 ± 2.0
Creatinine (mg/dL)	8.27 ± 0.32	8.32 ± 0.38
Total cholesterol	159.0 ± 6.0	155.5 ± 5.4
LDL-C (mg/dL) *n* = 34	83.1 ± 4.1	80.3 ± 3.8
HDL-C (mg/dL)	47.1 ± 2.8	48.8 ± 2.9
Triglyceride (mg/dL) *n* = 33	120.7 ± 7.7	124.4 ± 8.9
GA (%)	21.9 ± 0.6	22.7 ± 0.9
CPR (ng/mL) *n* = 32	9.39 ± 0.89	10.73 ± 0.94^*^
BG (mg/dL)	167.0 ± 7.1	162.7 ± 6.9
BNP (ng/mL) *n* = 32	405.5 ± 88.6	410.4 ± 90.8
oxLDL (U/L) *n* = 33	98.4 ± 5.1	91.6 ± 5.2
hsCRP (mg/dL) *n* = 30	0.204 ± 0.056	0.187 ± 0.056
8OHdG (ng/mL) *n* = 17	0.344 ± 0.021	0.338 ± 0.031

^*^ *P* < 0.05 compared with before treatment

**Fig. 1:**
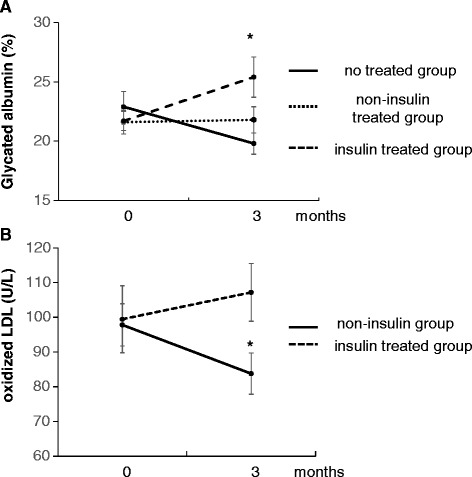
Levels of glycated albumin (GA) and oxidized low-density lipoprotein (oxLDL) in insulin-treated and non-insulin-treated groups measured before and after treatment. **a** GA levels in the non-treated group (*n* = 7), non-insulin-treated group (*n* = 16) and the insulin-treated group (*n* = 12). **b** Serum levels of oxLDL in the non-insulin group (*n* = 22) and insulin-treated group (*n* = 11). Data are the mean ± standard error of the mean. *P < 0.05 *vs.* values before treatment

Insulin is the most effective glucose-lowering agent, so switching from insulin to linagliptin increased GA levels in patients (Fig. 1A). Furthermore, reports have strongly suggested that hyperglycemia is a major mediator of oxidative stress in patients with diabetes through several mechanisms, such as stimulation of the polyol pathway [13], activation of protein kinase C [14], increased levels of glycated superoxide dismutase [15], and overproduction of superoxide in mitochondria [16]. Thus, to eliminate the glucose-lowering effects of insulin therapy, we further examined the efficacy of linagliptin in non-insulin-treated patients (*n* = 22).

To evaluate the anti-oxidative effects of linagliptin beyond its glucose-lowering effect, we re-analyzed data in patients who had not been treated with insulin previously as the “non-insulin group” (which included the non-treated group and non-insulin-treated group). Laboratory data (including GA) in non-insulin patients measured before and after switching to linagliptin are shown in Table 3, and oxLDL levels are shown in Fig. 1B. GA levels were not decreased by linagliptin treatment in the non-insulin-treated group, whereas levels of total cholesterol, LDL-C, and oxLDL were decreased significantly. Serum levels of total bilirubin were decreased significantly by linagliptin treatment. However, these changes were within the normal range and similar data were obtained for all patients, so these changes were not clinically significant.

**Table 3:** Comparison between the non-insulin group before and after treatment (*n* = 22)

Parameter	Before	After
BMI (kg/m^2^) *n* = 19	23.2 ± 1.0	23.3 ± 1.0
Systolic blood pressure (mmHg) *n* = 20	155.7 ± 6.3	162.3 ± 5.4
Diastolic blood pressure (mmHg) *n* = 20	78.7 ± 3.3	79.7 ± 3.0
Total protein (g/dL)	6.5 ± 0.1	6.6 ± 0.1
Albumin (g/dL)	3.7 ± 0.1	3.8 ± 0.1
AST (U/L)	14.9 ± 1.0	13.4 ± 1.2
ALT (U/L) *n* = 21	11.6 ± 1.4	10.1 ± 1.6
γ1.6 (U/L)	22.3 ± 3.0	23.2 ± 3.4
Total bilirubin (mg/dL) *n* = 17	0.35 ± 0.03	0.28 ± 0.02^*^
ALP (U/L) *n* = 21	251.0 ± 28.4	256.7 ± 25.8
LDH (U/L) *n* = 21	189.5 ± 6.0	179.2 ± 6.1
BUN (mg/dL)	54.5 ± 1.8	53.8 ± 2.3
Creatinine (mg/dL)	8.21 ± 0.34	8.28 ± 0.44
Total cholesterol (mg/dL)	162.6 ± 8.2	149.6 ± 6.9^*^
LDL-C (mg/dL) *n* = 21	83.1 ± 5.9	75.8 ± 5.0^*^
HDL-C (mg/dL)	46.9 ± 3.4	47.5 ± 3.5
Triglyceride (mg/dL) *n* = 21	119.7 ± 9.4	121.2 ± 11.9
CPR (ng/mL) *n* = 19	10.62 ± 1.20	11.56 ± 1.32
BG (mg/dL)	163.7 ± 9.2	162.7 ± 8.1
GA (%)	22.0 ± 0.8	21.2 ± 0.8
BNP (ng/mL) n = 21	429.2 ± 98.5	360.0 ± 79.3
hsCRP (mg/dL) *n* = 20	0.178 ± 0.046	0.193 ± 0.073
8OHdG (ng/mL) *n* = 12	0.356 ± 0.022	0.355 ± 0.041

^*^ *P* < 0.05 compared with before treatment

Abbreviations are the same as shown for Table 2

Other incretin therapies have also been reported to have anti-oxidative-stress effects [17]. To ascertain if lowered levels of oxLDL could be a characteristic effect of linagliptin, we compared oxLDL levels in patients according to whether or not they had been treated with incretin. Because of the small number of patients, we could not observe a significant change. oxLDL levels were decreased in eight incretin-treated patients (99.4 ± 8.2 to 90.3 ± 6.5 U/L) and 27 non-incretin-treated patients (98.0 ± 6.0 to 92.0 ± 6.3 U/L), independent of GA levels (incretin-treated: 21.8 ± 1.4 % to 21.7 ± 1.5 %; non-incretin-treated: 21.9 ± 0.7 % to 23.0 ± 1.0 %).

## Discussion

The present study demonstrated that linagliptin decreased serum levels of oxLDL in patients with type 2 diabetes undergoing HD independent of its glucose-lowering effect. LDL-C is readily oxidized in patients with diabetes [18]. In addition, oxLDL is an important factor for predicting cardiovascular disease in patients with diabetes [19]. Oxidative stress is increased if acute fluctuations of glucose levels occur (e.g., postprandial hyperglycemia). A positive interrelation between the mean amplitude of glucose excursion, urinary levels of 8-iso prostaglandin F_2α_, and a marker of oxidative stress has been reported [20]. DPP-4 inhibitors mainly target postprandial hyperglycemia and glucose fluctuations [21]. Therefore, we suggest that linagliptin decreases oxidative stress by inhibiting hyperglycemia and glucose fluctuations (though the latter parameter was not evaluated in the present study). Serum levels of active GLP-1 have been shown to be increased significantly in linagliptin-treated patients with type 2 diabetes undergoing HD [22]. In addition, the GLP-1 receptor agonist liraglutide has been demonstrated to decrease oxidative stress independent of its glucose-lowering effect [23]. However, we did not measure serum levels of active GLP-1 in the present study. In patients with diabetes, lowering levels of active lipoprotein lipase (which is an insulin-dependent enzyme) promotes postprandial hypertriglyceridemia and leads to increased levels of small, dense LDLs that are oxidized readily. DPP-4 inhibitors improve postprandial hypertriglyceridemia [24], so DPP-4 inhibitors could decrease oxLDL levels by increasing the size and density of LDL-C.

Furthermore, we speculate that the DPP-4 inhibitor linagliptin decreases oxidative stress directly. Eight patients received incretin therapy (Table 1) before switching to linagliptin treatment. In these patients, linagliptin caused a decrease in oxLDL levels from 99.4 ± 8.2 to 90.3 ± 6.5 U/L, suggesting that linagliptin exerts anti-oxidative-stress effects beyond those mediated by incretin.

Linagliptin is a unique, biliary-excreted DPP-4 inhibitor with anti-oxidative-stress effects based upon its xanthine structure [25], long half-life, and widespread distribution in tissues [26]. These characteristics contribute to decreased oxLDL levels. However, we did not observe reduction of oxLDL levels upon linagliptin treatment in insulin-treated patients, probably because GA levels were increased after switching from insulin to linagliptin. This finding suggests that excess hyperglycemia might elicit more powerful oxidative stress than the anti-oxidative effects of linagliptin. Changes in oxLDL levels observed in the present study were within the normal range. However, a recent report suggested that oxLDL > 48 U/L is associated with cardiovascular risk factors [27]. Hence, we believe that this reduction in oxLDL levels could be clinically meaningful. In addition, we have reported that linagliptin decreases urinary levels of 8OHdG in non-diabetic mice after vascular injury [11]. Therefore, in the present study, we measured plasma levels of 8OHdG, but differences in levels detected before and after treatment were not observed. The different results between levels of oxLDL and 8OHdG might be caused by the characteristics of markers of oxidative stress. Several studies have reported that linagliptin reduces levels of the lipid markers of oxidative stress [28, 29], but there are several nucleic-acid markers. Linagliptin is soluble in lipids, so it might be predisposed to lipid oxidation. Furthermore, using an animal model, we have observed reductions in urinary levels of 8OHdG upon linagliptin treatment [11]. However, the short half-life of 8OHdG in serum might have complicated detection of changes in its levels upon linagliptin treatment in the present study.

GA levels were not changed significantly by switching to linagliptin in the non-insulin-treated group. DPP-4-inhibitor monotherapy has been shown to decrease the GA level by 2.9 % in drug-naïve Japanese patients with type 2 diabetes undergoing HD [30, 31]. However, we did not observe reductions in GA levels (probably because of the switch to linagliptin treatment) but GA levels were increased greatly in the insulin-treated group.

Chaykovska *et al.* reported that linagliptin decreases cardiac gene expression of BNP in 5/6-nephrectomized rats [32]. However, though there were no changes in serum levels of BNP in insulin-treated patients, levels decreased in non-insulin-treated patients (though this difference was not significant). When proBNP in myocardial cells is secreted into the bloodstream, it is broken down into NT-proBNP and BNP_1–32_. DPP-4 cleaves BNP_1–32_ to produce BNP_3–32_ [33]_,_ which has low bioactivity. Consequently, the diuretic and tissue-protective effects of BNP_3–32_ are weaker than those of BNP_1–32_.

Levels of LDL-C and total cholesterol were decreased significantly in the non-insulin-treated group. In clinical trials, the DPP-4 inhibitor sitagliptin was also shown to decrease LDL-C levels in Japanese patients with type 2 diabetes [34], suggesting that this lipid-lowering effect is characteristic of this class of compound. This effect was also observed in patients undergoing HD in the present study.

The present study had limitations. First, the number of patients was relatively low, and the number of patients in the subgroup analysis was also too small. Second, our study was not a head-to-head comparison. Finally, it was not possible to evaluate parameters for some patients. Hence, further work is warranted to validate our findings.

## Conclusion

Linagliptin lowered serum levels of oxLDL in patients with type 2 diabetes who did not switch from insulin treatment and who were undergoing HD. This action of linagliptin was independent of its glucose-lowering effect.

## Abbreviations

8isoPGF_2α_: 8-iso prostaglandin F_2α_
8OHdG: 8-hydroxy-2′-deoxyguanosine
γGTP: γ-glutamyl transpeptidase
AGE: Advanced glycation end-product
AST: Aspartate aminotransferase
ALT: Alanine aminotransferase
ALP: Alkaline phosphatase
BG: Blood glucose
BNP: B-type natriuretic peptide
BUN: Blood urea nitrogen
DPP-4: Dipeptidyl peptidase-4
GA: Glycated albumin
GLP-1: Glucagon-like peptide-1
HDL-C: High-density lipoprotein-cholesterol
hsCRP: High-sensitivity C-reactive protein (hsCRP)
LDH: Lactate dehydrogenase
LDL-C: Low-density lipoprotein-cholesterol
oxLDL: Oxidized low-density lipoprotein
